# An indocyanine green-based liquid biopsy test for circulating tumor cells for pediatric liver cancer

**DOI:** 10.1097/HC9.0000000000000435

**Published:** 2024-05-10

**Authors:** Andres F. Espinoza, Pavan Kureti, Roma H. Patel, Susan L. Do, Saiabhiroop R. Govindu, Bryan W. Armbruster, Martin Urbicain, Kalyani R. Patel, Dolores Lopez-Terrada, Sanjeev A. Vasudevan, Sarah E. Woodfield

**Affiliations:** 1 Pediatric Surgical Oncology Laboratory, Michael E. DeBakey Department of Surgery, Divisions of Pediatric Surgery and Surgical Research, Texas Children’s Surgical Oncology Program, Texas Children’s Liver Tumor Program, Dan L. Duncan Cancer Center, Baylor College of Medicine, Houston, Texas, USA; 2 Department of Pathology and Immunology, Baylor College of Medicine, Texas Children’s Department of Pathology, Houston, Texas, USA

## Abstract

**Background:**

Hepatoblastoma and HCC are the most common malignant hepatocellular tumors seen in children. The aim of this study was to develop a liquid biopsy test for circulating tumor cells (CTCs) for these tumors that would be less invasive and provide real-time information about tumor response to therapy.

**Methods:**

For this test, we utilized indocyanine green (ICG), a far-red fluorescent dye used clinically to identify malignant liver cells during surgery. We assessed ICG accumulation in cell lines using fluorescence microscopy and flow cytometry. For our CTC test, we developed a panel of liver tumor-specific markers, including ICG, Glypican-3, and DAPI, and tested it with cell lines and noncancer control blood samples. We then used this panel to analyze whole-blood samples for CTC burden with a cohort of 15 patients with hepatoblastoma and HCC and correlated with patient characteristics and outcomes.

**Results:**

We showed that ICG accumulation is specific to liver cancer cells, compared to nonmalignant liver cells, non-liver solid tumor cells, and other nonmalignant cells, and can be used to identify liver tumor cells in a mixed population of cells. Experiments with the ICG/Glypican-3/DAPI panel showed that it specifically tagged malignant liver cells. Using patient samples, we found that CTC burden from sequential blood samples from the same patients mirrored the patients’ responses to therapy.

**Conclusions:**

Our novel ICG-based liquid biopsy test for CTCs can be used to specifically detect and quantify CTCs in the blood of pediatric patients with liver cancer.

## Graphical abstract

**Figure:**
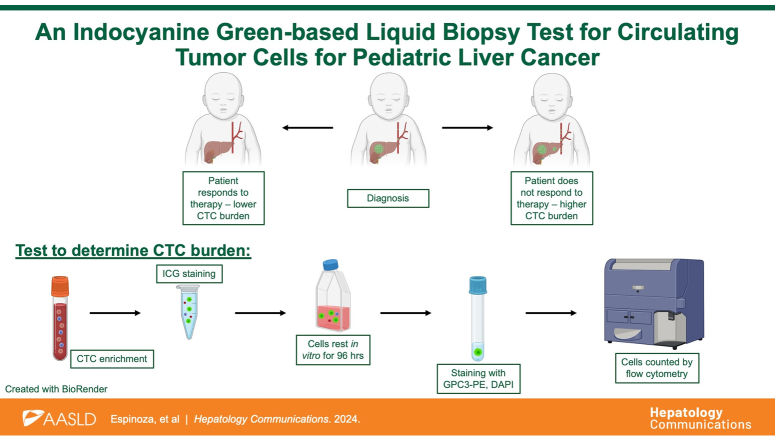

## INTRODUCTION

Hepatoblastoma (HB) is the most common malignant hepatocellular tumor seen in children and generally affects individuals younger than 5 years of age with <50% overall survival (OS) in patients who present with multifocal, metastatic, or treatment-refractory disease.^1–3^ Notably, HB has the fastest rising incidence of all pediatric solid tumors,^4^ potentially due to its association with premature birth, maternal environmental exposures, and cancer predisposition syndromes.^5,6^ Current frontline therapy for HB includes aggressive surgery, either resection or OLT, with cisplatin/doxorubicin-based chemotherapy. Despite receiving intense treatment, children with high-risk HB attain <50% 5-year event-free survival and suffer many permanent toxicities related to therapy.^1,7–10^ Pediatric HCC has a dismal 5-year OS rate of ~28%,^11^ although patients with resectable disease achieve a much better OS rate of 70%–80%.^12^ Medical therapy for pediatric HCC is mainly limited to a chemotherapy regimen based on cisplatin and doxorubicin, although the kinase inhibitor sorafenib has also been tested.^13^ The most meaningful impact on HCC outcomes has been in the field of interventional radiology, where chemo- or radio-embolization can be used to prolong survival.^14^ Fibrolamellar carcinoma (FLC) is a very rare subtype of HCC that occurs in adolescents and young adults, with a 5-year OS rate of ~50% for children.^15,16^ Surgical resection is the only curative treatment for this disease, and there are no standard-of-care systemic therapies for FLC.^15^

Generally, pediatric patients with liver tumors are initially diagnosed by imaging and assessment of serum levels of alpha-fetoprotein (AFP), followed by biopsy for tissue diagnosis and subtype classification. Treatment plans are individualized based on patient age, histopathological and imaging information, and blood AFP levels.^2^ During therapy, longitudinal clinical monitoring of patients is completed with imaging and blood AFP levels. Patients are deemed disease-free when tumors are no longer visible by current imaging modalities and AFP levels have dropped to normal levels.

Unfortunately, current strategies for matching individual cases to effective therapeutic strategies are suboptimal. HB and pediatric HCC are very heterogeneous tumors, and, with limited tumor material from biopsies, not all tumor subclones may be captured. In addition, AFP levels can be misleading, as some tumors do not secrete AFP. In addition, infants, particularly premature babies, may have high AFP levels because of the ongoing development of the liver.^17^ For children, repeated imaging can increase future oncologic risk and cause neurological damage as they are exposed to radiation and repeat sedation. Standard imaging modalities do not have the resolution required for the detection of individual tumor cells, and patients may harbor one or a few tumor cells at the end of therapy.^18^ Clinical tests with the ability to detect “minimal residual disease” in the form of circulating tumor cells (CTCs) may aid in identifying such patients before they progress to visible or detectable recurrence or metastases.

Liquid biopsy assays for CTCs circumvent many of these challenges. A liquid biopsy involves a peripheral whole-blood draw, followed by an assessment of the blood for tumor material, including CTCs and circulating tumor DNA. Blood sampling for tumor cells provides a readout of the most aggressive clones present that are already in the process of dissemination.^19^ In addition, sequential sampling of CTCs can provide information about the evolution of tumor cells as they change in response to therapies.^19^ These cells provide a precise evaluation of the real-time state of the tumor at diagnosis or as the patient undergoes therapy.^19^ The major challenge in developing liquid biopsy assays for CTCs is unambiguously identifying these cells in the blood; these tumor cells are very rare in the blood, with ~1 in 10^6^ leukocytes.^20^ Both marker-based and physical property–based protocols for isolating CTCs have been validated with only 1 platform, the CELLSEARCH platform, clinically approved for use.^21^ The key to marker-based protocols is the use of markers that are common to all tumor cells, even if these cells evolve as they enter the blood and disseminate.

To address these challenges in the development of a liquid biopsy test for CTCs, we harnessed the power of indocyanine green (ICG), a far-red fluorescent dye that accumulates in tumor cells of a liver origin. ICG is currently validated for clinical use during surgery to identify liver tumor cells throughout the body, including cells that represent intrahepatic primary liver tumors or extrahepatic disease, such as lung metastasis.^22–28^ It is not fully understood why liver tumor cells accumulate ICG, but is thought to involve endocytic uptake of ICG into tumor cells, which have higher endocytic activity than normal cells, followed by delayed clearance due to trapping in the membrane traffic system because of disrupted tight junctions in tumor cells.^29^ This mechanism may also involve the solute carrier transporter OATP1B3.^30^ Although ICG has been most widely used with malignant liver tumors, some reports show that ICG accumulates in other tumor cells, such as lung,^31,32^ osteosarcoma,^33^ and gastric cancer,^34^ presumably due to similar increased endocytic activity, disrupted tight junctions, and OATP1B3. To validate ICG^+^ cells as liver tumor cells, we chose a second marker of HB and HCC tumor cells, Glypican-3 (GPC3). GPC3 was shown to be elevated in a study of 60 HB tumor samples and negative in adjacent benign liver parenchyma, illustrating that this marker is highly expressed in HB cells.^35^ In addition, GPC3 is overexpressed in HCC.^36^ This specificity of GPC3 for hepatocellular cancer cells has led to the development of GPC3-targeting chimeric antigen receptor T-cell trials that are currently being evaluated in clinical trials enrolling pediatric and adult patients with liver tumors.

In this study, we describe a novel liquid biopsy assay for pediatric liver cancer CTCs. This test is based on the unambiguous identification of cells that express 3 markers, ICG, GPC3, and DAPI with both standard and imaging flow cytometry. Importantly, this test can be used for the most common pediatric primary tumors of the liver, HB and HCC, including the FLC subtype of HCC.

## METHODS

### Cells and culture conditions

The HepG2 (from a patient aged 15 y), Huh-6 (from a patient aged 1 y), HepRG (patient age not specified), Hep3B (from a patient aged 8 y), and A549 (from a patient aged 58 y) cell lines were commercially acquired (HepG2 [HB-2065], Hep3B [HB-8064], and A549 [CRM-CCL-185]: American Type Culture Collection [ATCC]; Huh-6: Riken Cell Bank; HepRG: HPRGC10, Invitrogen). The SH-SY5Y (from a patient aged 4 y), Huh-7 (from a patient aged 57 y), and 293T (embryonic) cell lines were generously provided by Dr Jianhua Yang (Baylor College of Medicine). All widely available cell lines were grown in Eagle’s Minimum Essential Medium (EMEM, Lonza) supplemented with 10% heat-inactivated fetal bovine serum (SAFC Biosciences), 2 mM glutamine (Invitrogen), and 100 units/mL streptomycin/penicillin (Invitrogen).

The HB17 cell line (from a patient aged 1 y) was developed in our laboratory from a patient-derived xenograft tumor.^37^ Cells from the tumor were grown in vitro initially on Matrigel (354230, Corning) in Hepatocyte Culture Medium (CC-3198, Lonza). After 40 passages, the cells were transitioned to standard tissue culture plasticware without Matrigel.

All cells were grown at 37°C in 5% CO_2_. All cells were validated with short tandem repeat DNA profiling yearly. Profiles of widely available cell lines were compared to a database of cell line profiles. The HB17 profile was compared to a profile generated by short tandem repeat DNA profiling of the paired primary patient sample. All cells were also tested for mycoplasma (MycoAlert, Lonza) every year.

### ICG

ICG was obtained as a powder (United States Pharmacopeia) diluted to 2 mg/mL in water, and then added at 25 μM to cells and patient samples.

### Fluorescent antibodies and other markers

A GPC3 antibody conjugated to the phycoerythrin (PE) fluorophore (clone 1G12 + GPC3/863, NBP2-47763PE, Novus Biologicals) was used with immunofluorescence and flow cytometry experiments as described at a dilution of 1:100. For living cells, DAPI (cat. no. R37605, NucBlue Live Cell Stain ReadyProbes, Invitrogen) was used according to the manufacturer’s protocol for microscopy experiments. For fixed cells, DAPI (cat. no. R37606, NucBlue Fixed Cell Stain ReadyProbes, Invitrogen) was used for flow cytometry experiments at a dilution of 2 drops per 1 mL.

### Standard flow cytometry

Samples were analyzed with flow cytometry on a standard Symphony instrument (BD Biosciences) using a 637 nm laser to excite ICG and a 780/60 bandpass filter to detect it, a 561 nm laser to excite PE and a 586/15 bandpass filter to detect it, and a 405 nm laser to excite DAPI and a 431/28 bandpass filter to detect it. All cell line samples were gated as follows: (1) forward scatter (FSC)-area versus side scatter (SSC)-area to identify cells; (2) FSC-area versus FSC-width to identify single cells; (3) SSC-height versus SSC-width to identify single cells; (4) histogram of DAPI-area to identify DAPI^+^ enucleated cells; (5) histogram of APC-Cy7-area to identify ICG^+^/DAPI^+^ cells; and (6) histogram of PE-area to identify GPC3-PE^+^/ICG^+^/DAPI^+^ cells (if applicable). All patient samples were gated as follows: (1) FSC-area versus FSC-width to identify single cells; (2) SSC-height versus SSC-width to identify single cells; (3) histogram of DAPI-area to identify ICG^+^/DAPI^+^ enucleated cells; (4) histogram of APC-Cy7-area to identify ICG^+^ cells; and (5) histogram of PE-area to identify GPC3-PE^+^/ICG^+^/DAPI^+^ cells. All samples were run with appropriate positive and negative controls as described. All patient samples were run with the following HepG2 and A549 positive and negative controls: (1) HepG2 negative; (2) HepG2 ICG^+^/GPC3^+^/DAPI^+^; (3) A549 negative; and (4) A549 GPC3^+^/DAPI^+^. All data were analyzed with FlowJo version 10.6.2 (Becton Dickinson). For patient samples, collection was performed on the entirety of the sample.

### Imaging flow cytometry

Samples were analyzed with flow cytometry on an Amnis Imagestream X MKII (Luminex) equipped with 405, 488, 561, 633, and 785 nm scatter lasers. Collection was performed on the entirety of each sample. Objects were analyzed and gated using IDEAS software 6.3.23.0 as follows: (1) cells were gated using Aspect Ratio and Area parameters for the brightfield channel. First, focused cells were gated using the Gradient_RMS parameter for Channel 1 (Brightfield). Second, single cells were gated using the Area versus Aspect Ratio for Channel 1 (Brightfield). (2) DAPI^+^ single cells were gated with a histogram of the Intensity of Channel 7 (CCR7/BV421) with the single-cell population. (3) DAPI^+^/ICG^+^/GPC3^+^ single cells were gated with a scatter plot of the Intensity of Channel 12 (APC-Cy7/APC-ef780/Live Dead NIR) versus the Intensity of Channel 3 (PE) with the DAPI^+^ population. Representative images used for figures were exported from the IDEAS image gallery as .tif files and inserted into the manuscript. Raw data files are available upon request. All samples were run with appropriate HepG2 and A549 positive and negative controls: (1) HepG2 negative; (2) HepG2 ICG^+^/GPC3^+^/DAPI^+^; (3) HepG2 ICG^+^; (4) HepG2 GPC3^+^; (5) HepG2 DAPI^+^; (6) A549 negative; and (7) A549 GPC3^+^/DAPI^+^.

### Microscopy

Brightfield and fluorescent images of cells were taken on a BZ-X710 All-in-One Fluorescence Microscope (Keyence) at the indicated magnifications and scales with ICG and DAPI filters. The objective used was a Nikon 20X PlanFluor objective with a numerical aperture of 0.45. The microscope and acquisition software used was the standard for this microscope. All images were taken at room temperature. Cells were alive in cell culture media and tagged with mCherry, ICG, and DAPI as indicated.

### Patient samples

This study was a prospective study of 16 patients who were diagnosed with malignant or benign primary liver tumors. Samples were collected during a time period of almost 2 years from patients after informed consent was obtained from their parents or guardians through an Institutional Review Board (IRB)–approved blood collection protocols H-38834 and H-49313. All experiments on patient samples were performed in compliance with the Helsinki Declaration and approved by the Baylor College of Medicine IRB.

### Processing of human whole-blood samples

Samples were depleted of hematopoietic cells with the RosetteSep CD45 depletion kit (depletes with antibodies for CD45, CD66, and Glycophorin A, cat. no. 15122, Stem Cell Technologies) according to the protocol with SepMate-15 tubes (cat. no. 85415, Stem Cell Technologies) and Lymphoprep (cat. no. 1114544, Alere Technologies). The remaining cells were then washed with ACK lysis buffer (cat. no. 118-156-101, Quality Biological) to lyse the remaining red blood cells. Samples were then incubated with 25 μM ICG for 1 hour at 37°C. Some samples collected from hepatectomy procedures may have been exposed to ICG as part of the preoperative administration of ICG for surgery. However, this time point would have been ~168 hours before the final collection of cells and would not have influenced the final ICG positivity of cells. After ICG incubation, cells were kept in culture for 96 hours in a complete hepatocyte basal medium (cat. no. CC-3198, Lonza). Cells were then fixed in 4% paraformaldehyde (cat. no. 15710, Electron Microscopy Sciences) for 30 minutes and stained with GPC3-PE antibody (1:100) for 30 minutes. Cells were incubated with DAPI (see above) before flow cytometry experiments.

### GPC3 IHC

Patient primary tumor and lung metastasis samples were fixed in 10% formalin, embedded in paraffin, and mounted on glass slides in the Texas Children’s Hospital clinical pathology laboratory as part of the routine clinical care of these patients. Slides were stained with Glypican-3 (1G12, Cell Marque). Stained slides were then scanned with a Leica Aperio AT2 slide scanner. The slides were preprocessed to select the bounding area and focus points for each slide. After scanning, the images were saved on a secure hospital server. The GPC3 images shown in the manuscript are representative of the entire samples and were selected from the whole scanned area using QuPath version 0.4.3. GPC3 histology was quantified by a pathologist (Kalyani R. Patel) with a standard scoring system of Intensity (I) + Extent (E)=T (Total). Intensity was scored as 0 (absent), 1 (weak), 2 (intermediate), or 3 (strong). Extent was scored as 0 (0), 1 (>0% to <25%), 2 (>25% to <50%), 3 (>50% to <75%), or 4 (>75% to 100%). This information is shown in Table 2, along with the histology of each tissue sample (also assessed by a pathologist [Kalyani R. Patel]).

### Patient ICG

ICG was utilized during hepatectomy and metastasectomy for pediatric liver tumors as described^22^ at Texas Children’s Hospital. ICG images of primary samples shown in Figures 4, 5, and 6 were obtained from electronic medical records.

### Patient characteristics

We reviewed electronic medical records for the following patient-specific information shown in Table 1: (1) diagnosis, (2) pretreatment extent of disease (PRETEXT) stage,^38^ (3) Children’s Oncology Group (COG) stage,^7^ (4) risk group,^2^ (5) the presence of multifocal disease in the liver, (6) the presence of vascular invasion, (7) the presence of metastasis, and (8) ICG positivity. We reviewed electronic medical records for the following sample-specific information shown in Table 2: (1) time point in care when the sample was received, (2) whether chemotherapy had been received by the patient at that time point, (3) AFP level at that time point, and (4) disease burden at that time point. Disease burden was determined by assessing available clinical data to estimate whether tumor burden was increasing or decreasing following therapy. Diagnostic biopsy samples are considered separate from other samples for increasing or decreasing burden. We were blinded to CTC burden when we assessed disease burden. CT images shown in Figures 4, 5, and 6 were taken as part of routine clinical care and were extracted from electronic medical records for this manuscript.

**TABLE 1:** Patient data

Patient ID	Diagnosis	Age (y)	Sex	PRETEXT stage	COG stage	Risk	Multifocal	VI	Metastasis	ICG+
HB70/110	HB	2	F	1	1	VLR	N	N	N	Y
HB73	HB	3	M	4	4	HR	Y	Y	Y	NA
HB77	HB	1	M	2	1	LR	N	Y	N	Y
HB78	HB	1	M	3	1	IR	Y	N	N	NA
HB80	HB	1	F	4	1	HR	N	N	Y	Y
HB99	HB	5	M	3	4	HR	N	Y	Y	Y
HB102/106	HB	8	M	3	2	IR	Y	Y	N	Y
HB103/113	HB	5	F	4	4	HR	Y	Y	Y	Y
HB108	HB	1	M	4	3	IR	Y	Y	N	NA
HB109	HB	0.42	F	2	1	VLR	N	Y	N	NA
HB112	HB	1	F	3	2	HR	N	Y	Y	Y
HB134	HB	1	M	3	1	IR	N	Y	N	Y
HB83/114/116	HCC	7	F	4	4	HR	Y	Y	Y	Y
HB105	FLC	21	M	3	2	HR	Y	Y	Y	Y
HB119	FLC	8	M	4	NA	HR	Y	Y	N	NA
HB71	MH	2	F	2	NA	NA	N	N	N	NA

*Note*: Patient age shown at the time of the first sample. NA for ICG^+^ indicates that ICG was not used during surgery so information about ICG accumulation is not available.

Abbreviations: COG, Children’s Oncology Group; FLC, fibrolamellar carcinoma; HB, hepatoblastoma; HR, high-risk; ICG, indocyanine green; LR, low risk; MH, mesenchymal hamartoma; NA, not available; PRETEXT, pretreatment extent of disease; VI, vascular invasion; VLR, very low risk.

**TABLE 2:** Sample data

Patient ID	Diagnosis	Sample	Time/procedure	Chemotherapy received	AFP	Disease burden	CTC count (cells/mL)—standard	CTC count (cells/mL)—imaging	Histology	GPC3 score of clinical sample (I+E=T)
HB70/110	HB	HB70	Hepatectomy	N	978	Increasing	31.80		Mixed epithelial (mitotically inactive fetal, embryonal), mesenchymal HB	Epithelial 3+4=7
		HB110	Clinic	Y	3.5	Decreasing		11.00		Mesenchymal 1+1=2
HB73	HB	HB73	Biopsy	N	207,000	Increasing	28.00		Epithelial (mitotically inactive fetal, embryonal) HB	2.5+4=7
HB77	HB	HB77	Hepatectomy	Y	2931	Decreasing	26.62	36.47	Epithelial (mitotically inactive fetal, embryonal), mesenchymal with teratoid	Epithelial 3+4=7
HB78	HB	HB78	Biopsy	N	161	Increasing	2.25	5.00	Epithelial (mitotically inactive fetal) HB	2+2=4
HB80	HB	HB80	Hepatectomy	Y	40	Decreasing	75.50	79.00	Mixed epithelial (mitotically inactive fetal, embryonal), mesenchymal, teratoid HB	Epithelial 2+4=6
HB99	HB	HB99	Metastasectomy	Y	14	Decreasing	0.00		Epithelial (mitotically inactive fetal, embryonal) HB	2+3=5
HB102/106	HB	HB102	TARE	Y	>207,000	Increasing	7.03	0.00	HCN, NOS	2+2=4
		HB106	Hepatectomy	Y	>207,000	Increasing	88.67	9.33		
HB103/113	HB	HB103	Metastasectomy	Y	52.6	Increasing	0.00	0.00	Epithelial HB	3+4=7
		HB113	Metastasectomy	Y	601	Increasing	21.00	18.00		
HB108	HB	HB108	Transplant	Y	195	Decreasing	7.50	11.50	Epithelial HB	Not available
HB109	HB	HB109	Hepatectomy	N	102,000	Increasing	254.00	231.00	Epithelial (mitotically inactive fetal, embryonal) HB	2+1=3
HB112	HB	HB112	Hepatectomy	Y	405	Decreasing	6.00	0.00	Mixed epithelial (mitotically inactive fetal), mesenchymal, teratoid HB	Epithelial 3+4=7
HB134	HB	HB134	Hepatectomy	Y	101	Decreasing	0.00		Epithelial, mesenchymal HB	Not available
HB83/114/116	HCC	HB83	TARE	Y	>207,000	Increasing	7.50	10.00	HCC	3+4=7
		HB114	Hepatectomy	Y	10.5	Decreasing	0.20			
		HB116	Metastasectomy	Y	2.1	Decreasing	8.00			
HB105	FLC	HB105	Metastasectomy	Y	NA	Increasing	10.50		FLC	0+0=0
HB119	FLC	HB119	Biopsy	N	<.8	Increasing	54.04		FLC	0+0=0
HB71	MH	HB71	Hepatectomy	N	33		1.20		MH	0+0=0

*Note*: CTC burden shown is the number of DAPI^+^/ICG^+^/GPC3^+^ cells measured by the indicated flow cytometry instrument.

Abbreviations: AFP, alpha-fetoprotein; CTC, circulating tumor cell; FLC, fibrolamellar carcinoma; GPC3, Glypican-3; HB, hepatoblastoma; HCN, hepatocellular neoplasm; MH, mesenchymal hamartoma; NA, not available; NOS, not otherwise specified; TARE, transarterial radioembolization.

The data generated in this study are available upon request from the corresponding author.

## RESULTS

### ICG selectively accumulates in liver cancer cells

**FIGURE 1:**
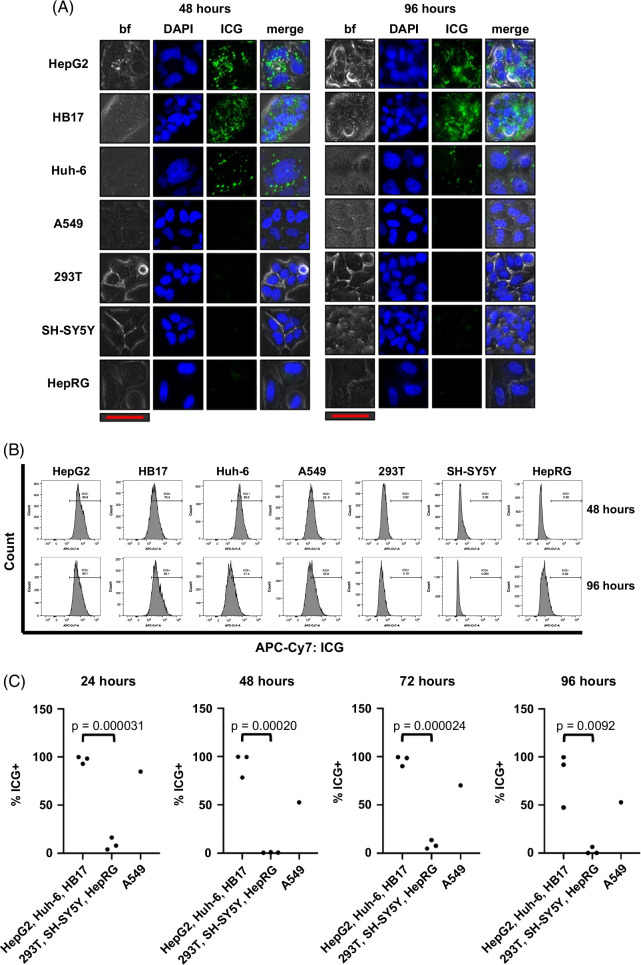
ICG selectively accumulates in liver cancer cells. Cells stained with 25 μM ICG for 1 hour and then imaged or analyzed by flow cytometry 48 and 96 hours after incubation. There is clear ICG accumulation in the HepG2, Huh-6, and HB17 HB tumor cells at these time points, while the nontumor HepRG cell line, the non-liver SH-SY5Y and A549 cell lines, and the non-liver and nontumor 293T cell line lack ICG. (A) Images of cells, which were also tagged with DAPI to show nuclei present in each image. The scale bar (red) represents 50 μm. Merge is bf, DAPI, and ICG images overlaid in 1 image. The exposure time is 5 seconds. (B, C) Histograms of the DAPI^+^/ICG^+^ population in each sample analyzed by flow cytometry shown in (B). Percent positive for ICG^+^ shown in (C) with statistical significance analyzed by Student *t* test (2-tailed). Abbreviations: bf, brightfield; ICG, indocyanine green.

We first sought to show that ICG selectively accumulates in liver cancer cells in vitro by fluorescence microscopy. We optimized the dosing of ICG and imaging timing to show positive ICG presence in liver tumor cells with accompanying negative signals in nontumor and non-liver cells. As shown in Supplemental Figure S1, http://links.lww.com/HC9/A872, after incubation with ICG for 1 hour at a dose of 25 µM, we saw clear ICG accumulation in the HepG2, Huh-6, and HB17 HB tumor cells 24 hours later (Supplemental Figure S1A, http://links.lww.com/HC9/A872). At the same dose and time points, we observed a lack of ICG in the nontumor HepRG cell line, the non-liver SH-SY5Y (neuroblastoma) and A549 (lung cancer) cell lines, and the non-liver and nontumor 293T cell lines (Supplemental Figure S1A, http://links.lww.com/HC9/A872). The results remained consistent at extended intervals of 48, 72, and 96 hours after incubation with ICG (Figure 1A and Supplemental Figure S1A, http://links.lww.com/HC9/A872). Notably, the HepG2 and HB17 cells appeared even brighter at the 72- and 96-hour postincubation intervals. At the same time points, we observed low or negative signals in the nontumor and non-liver cell lines (Figure 1A and Supplemental Figure S1A, http://links.lww.com/HC9/A872). We also tested ICG accumulation of HCC cell lines Hep3B and Huh-7 at the 96-hour postincubation time point and observed strong ICG signal in both cell lines (Supplemental Figure S2A, http://links.lww.com/HC9/A873).

This ICG positivity can also be analyzed by flow cytometry with APC-Cy7 fluorophore conditions. HepG2, HB17, and Huh-6 cells stained with 25 µM ICG and flowed 24, 48, 72, and 96 hours after incubation showed clear high APC-Cy7/ICG signal, whereas SH-SY5Y, 293T, and HepRG cells stained with the same concentration of ICG and analyzed by flow cytometry at the same time points showed a very low signal (Figures 1B, C and Supplemental Figure S1B, http://links.lww.com/HC9/A872). By flow cytometry, A549 cells showed intermediate ICG accumulation (Figures 1B, C and Supplemental Figure S1B, http://links.lww.com/HC9/A872). These data are further analyzed in Figure 1C, showing that malignant liver cells showed statistically significant higher levels of ICG fluorescence intensity at all 4 time points, compared to 293T, SH-SY5Y, and HepRG cells. We also found that HCC cell lines Hep3B and Huh-7 showed higher levels of ICG fluorescence intensity at the 96-hour postincubation time point by flow cytometry (Supplemental Figure S2B, http://links.lww.com/HC9/A873). Taken together, this study showed that ICG accumulation can be used to unambiguously identify tumor cells of a liver origin.

### ICG far-red fluorescent signal can be used to identify liver cancer cells in a mixed population of cells

**FIGURE 2:**
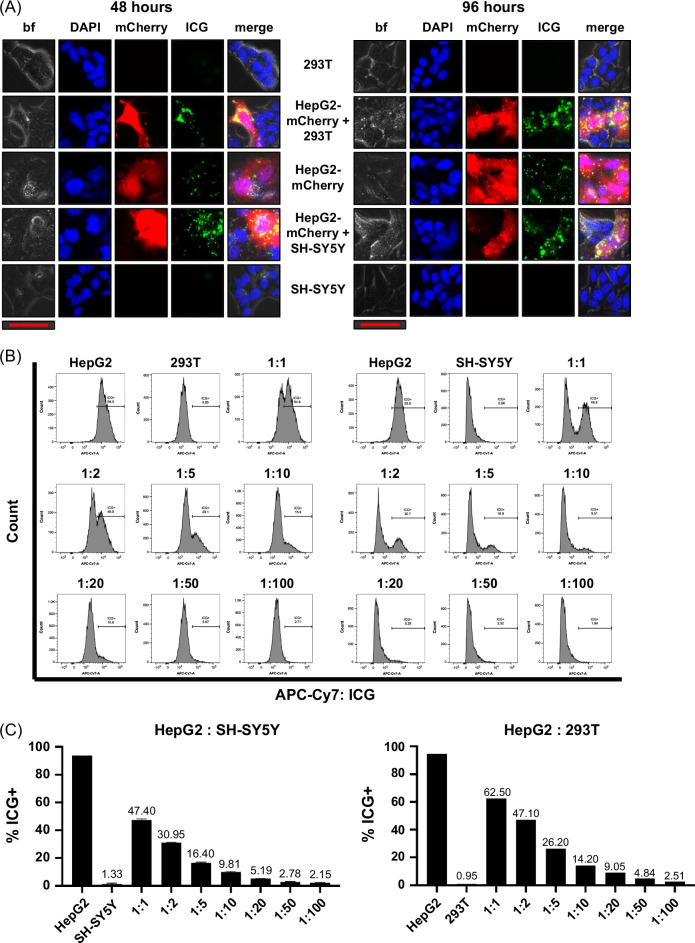
ICG can be used to identify liver cancer cells in a mixed population of cells. (A) We mixed HepG2 cells expressing mCherry (HepG2-mCherry) with either 293T or SH-SY5Y cells at a 1:1 ratio and then stained all cells with 25 µM ICG for 1 hour, followed by imaging at 48 and 96 hours after 1-hour incubation with ICG. Cells were also mixed with DAPI to stain the nuclei of cells present in each image. There is a clear positive ICG signal in the mCherry^+^ HepG2 cells while the 293T or SH-SY5Y cells in the same well remain negative. The scale bar (red) represents 50 μm. Merge is bf, DAPI, and ICG images overlaid in 1 image. The exposure time is 5 seconds for both 48- and 96-hour time points. (B, C) HepG2, 293T, and SH-SY5Y cells were stained with ICG for 1 hour and then analyzed by flow cytometry 96 hours after incubation. HepG2 cells were mixed with either 293T or SH-SY5Y cells at the indicated ratios. Histograms of the DAPI^+^/ICG^+^ population in each sample shown in (B) with a clear separation between the ICG^+^ HepG2 cells and ICG^−^ 293T or SH-SY5Y cells. Graphs in (C) show the percent ICG^+^ cells in each mixture, which corresponds to the ratio of HepG2 cells present in the mix. Abbreviations: bf, brightfield; ICG, indocyanine green.

We further verified that ICG positivity is specific to liver cancer cell lines and not secondary to staining conditions by analyzing mixed populations of cells by fluorescence microscopy. For these experiments, we used HepG2 cells expressing mCherry (HepG2-mCherry). We mixed HepG2-mCherry cells with either 293T or SH-SY5Y cells at a 1:1 ratio and then stained all cells with 25 µM ICG for 1 hour, followed by imaging at 48 and 96 hours after 1-hour incubation with ICG. Figure 2A showed a clear positive ICG signal in the mCherry^+^ HepG2 cells while the 293T or SH-SY5Y cells in the same well remained negative.

In addition, this ICG positivity can be used to selectively identify HB tumor cells mixed with 293T or SH-SY5Y cells by flow cytometry. We mixed HepG2 with 293T or SH-SY5Y cells that were both incubated with 25 µM ICG for 1 hour. We mixed them together at a range of ratios as indicated in Figures 2B, C and analyzed them by flow cytometry with APC-Cy7 fluorophore conditions to count ICG positivity at a single 96-hour time point after ICG incubation, the point at which we saw the greatest difference between positive and negative populations. The histograms in Figure 2B showed the separation of the 2 cell populations at both time points when the cells were mixed at the indicated ratios. The graphs in Figure 2C showed that the percent positive for ICG coincided with the ratio of HepG2 cells present in the mix.

### Unambiguous identification of liver tumor cells with a panel of ICG, GPC3, and DAPI

**FIGURE 3:**
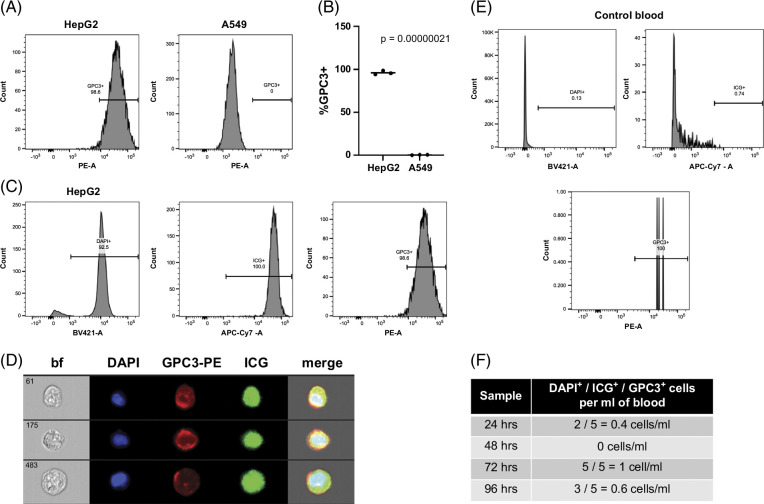
A marker panel of ICG, GPC3, and DAPI can be used to unambiguously identify liver tumor cells. (A, B) Flow cytometry for GPC3 with HepG2 positive control cells and A549 negative control cells shows the specificity of GPC3 for HepG2 cells that express this protein. Histograms of the DAPI^+^/GPC3^+^ population in each sample shown in (A). Percent DAPI^+^/GPC3^+^ for each sample graphed in (B) with statistical significance analyzed by Student *t* test (2-tailed). (C) Gating strategy for samples run with the DAPI/ICG/GPC3 panel. Single cells were first separated by gating with FSC and SSC as described in the Methods section. DAPI^+^/ICG^+^/GPC3^+^ cells were then separated and counted on histograms as follows: (1) DAPI^+^ enucleated cells, (2) ICG^+^ cells, and (3) GPC3^+^ cells. This is shown for a representative HepG2 sample. (D) Images of DAPI^+^/ICG^+^/GPC3^+^ HepG2 cells analyzed by the Amnis ImageStream instrument. (E) Gating strategy for control samples run with the DAPI/ICG/GPC3 panel. Single cells were first separated by gating with FSC and SSC as described in the Methods section. DAPI^+^/ICG^+^/GPC3^+^ cells were then separated and counted on histograms as follows: (1) DAPI^+^ enucleated cells, (2) ICG^+^ cells, and (3) GPC3^+^ cells. This is shown for the sample incubated with ICG for 96 hours. (F) DAPI^+^/ICG^+^/GPC3^+^ cells present in noncancer control blood samples. Blood samples were processed as described in the Methods section, and samples were stained with ICG for 1 hour, followed by resting in standard cell culture conditions in HBM for the indicated amount of time. Samples were then analyzed by flow cytometry on a BD Symphony instrument for numbers of DAPI^+^/ICG^+^/GPC3^+^ cells. All samples showed 1 cell or fewer per mL of blood. Abbreviations: bf, brightfield; FSC, forward scatter; GPC3, Glypican-3; HBM, hepatocyte basal medium; ICG, indocyanine green; SSC, side scatter.

To further validate the liver tumor origin of ICG^+^/DAPI^+^ cells, we added an antibody recognizing human GPC3, which is well established to be specifically expressed in HB.^35^ Shown in Figure 3A are histograms showing that 98.6% of DAPI^+^ HepG2 cells were also GPC3^+^. In contrast, all DAPI^+^ A549 cells were GPC3^-^. These data are further analyzed in Figure 3B, showing that liver tumor cells had a statistically significant increase in GPC3 expression compared to A549 cells. Multiple replicates of this experiment are plotted in Figure 3B, showing the reproducibility of this experiment.

We developed a panel to unambiguously identify pediatric liver tumor cells that include ICG, GPC3, and DAPI. After staining, we analyzed the samples by flow cytometry on a BD Biosciences standard flow cytometer and an Amnis ImageStream imaging flow cytometer, both with APC-Cy7, PE, and BV421 lasers. We chose to use the 96-hour time point after ICG incubation because ICG has been shown to be cleared from normal liver cells 3 days after exposure.^39,40^ We first tested this panel with the HB cell line HepG2 and the lung cancer cell line A549. Shown in Figure 3C is our gating strategy with our control HepG2 sample analyzed on the Symphony instrument. We used histograms for each channel to independently identify each population as follows: (1) DAPI^+^ cells with BV421-Area, (2) DAPI^+^/ICG^+^ cells with DAPI^+^ cells with APC-Cy7-Area, and (3) DAPI^+^/ICG^+^/GPC3^+^ cells with DAPI^+^/ICG^+^ cells with PE-Area. Importantly, with this strategy, 92.5% of HepG2 cells were DAPI^+^; of those, 100% were ICG^+^; and, of those, 98.6% were GPC3^+^, showing the specificity and sensitivity of the panel for identifying liver cancer cells (Figure 3C). Shown in Figure 3D are our control HepG2 cells flowed on the Amnis ImageStream instrument.

We then tested our panel on noncancer control whole-blood samples. We collected samples of 20 mL total of whole blood to analyze at 4 time points after collection and incubation with ICG: 24, 48, 72, and 96 hours. First, to decrease the contamination of immune cells in our downstream assays, we depleted samples for immune cells with the RosetteSep CD45 depletion kit (depleted with antibodies for CD45, CD66, and Glycophorin A). We then stained the remaining cells with our panel of ICG, GPC3-PE, and DAPI and analyzed the samples on the Symphony standard flow cytometer. Shown in Figure 3E is the gating strategy for the control blood samples, which is the same gating that was used with HepG2 cells (Figure 3C). As shown in Figure 3F, no samples showed more than 1 cell/mL of DAPI^+^/ICG^+^/GPC3^+^ cells.

### Unambiguous identification of CTCs with immune cell depletion and a panel of ICG, GPC3-PE, and DAPI

Twenty-one whole-blood samples from a cohort of 16 patients were analyzed with this panel of markers and standard and imaging flow cytometry. These patients represented diagnoses of HB, HCC, and FLC. One patient with a nonmalignant tumor, mesenchymal hamartoma, was used as a control. Characteristics of these patients are shown in Table 1. Counts for all of these samples on the standard and imaging flow cytometers, as well as individual sample characteristics, are shown in Table 2. Histological assessment and GPC3 positivity of a sample from each patient is also shown in Table 2. Importantly, correlating these CTC burdens with patient characteristics, including PRETEXT stage, COG stage, risk group, the presence of multifocal disease, the presence of vascular invasion, the presence of metastasis, chemotherapy at the time of sampling, AFP at the time of sampling, and disease burden, did not show any statistically significant correlations (Supplemental Figure S3, http://links.lww.com/HC9/A874). We observed a trend toward an association of higher CTC counts with diagnosis or with increasing disease burdens. For samples that had a CTC count on both the standard and imaging cytometers, we generally observed agreement with an average difference of 12.81 cells/mL and only 2 samples with a difference >10 cells/mL. As expected, the patient with mesenchymal hamartoma had a very low count of 1.2 cells/mL, similar to the noncancer control samples. For all patients, we assessed GPC3 expression and ICG accumulation to validate the use of our test based on GPC3 and ICG positivity.

**FIGURE 4:**
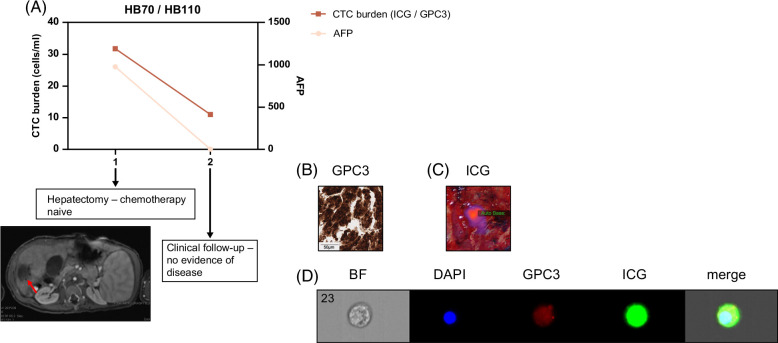
CTC burden in very-low-risk patient HB70/110. (A) We obtained samples at 2 time points during the patient’s course of treatment. We analyzed CTC burden after processing whole blood and tagging CTCs with ICG, GPC3, and DAPI, as described. We graphed CTC burden (cells/mL) and serum AFP levels, and both show a drop, correlating with the response of the patient to therapy. AFP was assessed by standard clinical tests. CT of primary tumor directly before hepatectomy with tumor indicated by a red arrow. (B, C) Validation of GPC3^+^ and ICG^+^ primary samples from patients. (B) Histology of primary patient tumor sample showing positivity of sample for GPC3. The scale bar represents 50 μm. (C) Near-infrared imaging of ICG^+^ primary tumor during hepatectomy. (D) Image of ICG^+^/GPC3^+^/DAPI^+^ CTC from Amnis ImageStream instrument. Abbreviations: AFP, alpha-fetoprotein; CTC, circulating tumor cell; GPC3, Glypican-3; ICG, indocyanine green.

**FIGURE 5:**
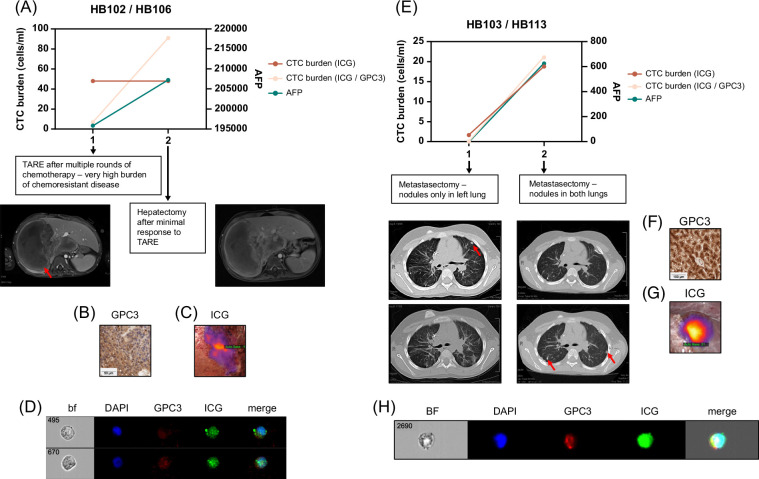
CTC burden in 2 high-risk patients, HB102/106 (A–D) and HB103/113 (E–H). (A) We obtained samples at 2 time points during the patient’s course of treatment. We analyzed CTC burden after processing whole blood and tagging CTCs with ICG, GPC3, and DAPI, as described. We graphed CTC burden (cells/mL) and serum AFP levels, and both show an increase, correlating with the patient not responding to therapy. AFP was assessed by standard clinical tests. DAPI^+^/ICG^+^/GPC3^+^ cell number shown is an average of counts by the standard and imaging flow cytometers for both samples. The DAPI^+^/ICG^+^ cell number shown was measured by the standard flow cytometer. CT scans from the time of TARE and the time of hepatectomy showed a minimal decrease in tumor (red arrow) size. (B, C) Validation of GPC3^+^ and ICG^+^ primary samples from patients. (B) Histology of primary patient tumor sample showing positivity of sample for GPC3. The scale bar represents 50 μm. (C) Near-infrared imaging of ICG^+^ primary tumor during hepatectomy. (D) Image of 2 ICG^+^/GPC3^+^/DAPI^+^ CTCs from Amnis ImageStream instrument. (E) We obtained samples at 2 time points during the patient’s course of treatment. We analyzed CTC burden after processing whole blood and tagging CTCs with ICG, GPC3, and DAPI, as described. We graphed CTC burden (cells/mL) and serum AFP levels, and both show an increase, correlating with the patient not responding to therapy. AFP was assessed by standard clinical tests. The DAPI^+^/ICG^+^/GPC3^+^ cell number shown is an average of counts by the standard and imaging flow cytometers for both samples. The DAPI^+^/ICG^+^ cell number shown was measured by the standard flow cytometer. CT images shown are the same slices in rows from 2 metastasectomy procedures, showing nodules in the left lung at the first time point (red arrow, left images) and new nodules present in both lungs (red arrows, right images) at the second time point. (F, G) Validation of GPC3^+^ and ICG^+^ primary samples from patients. (F) Histology of primary patient tumor sample showing positivity of sample for GPC3. The scale bar represents 100 μm. (G) Near-infrared imaging of ICG^+^ lung nodule during metastasectomy. (D) Image of ICG^+^/GPC3^+^/DAPI^+^ CTCs from Amnis ImageStream instrument. Abbreviations: AFP, alpha-fetoprotein; bf, brightfield; CTC, circulating tumor cell; GPC3, Glypican-3; ICG, indocyanine green; TARE, transarterial radioembolization.

**FIGURE 6:**
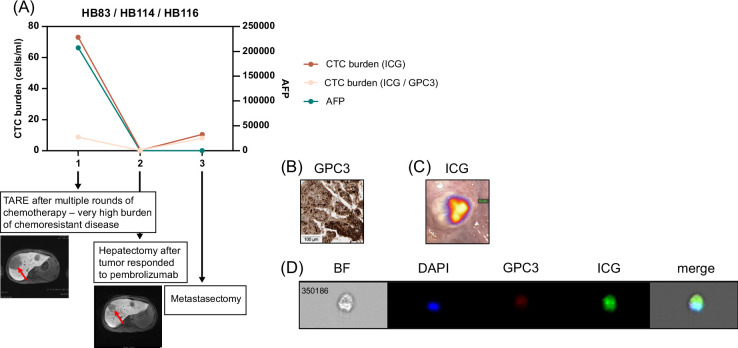
CTC burden in patient HB83/114/116 with HCC. (A) We obtained samples at 3 time points during the patient’s course of treatment. We analyzed CTC burden after processing whole blood and tagging CTCs with ICG, GPC3, and DAPI, as described. We graphed CTC burden (cells/mL) and serum AFP levels, and both show a drop, correlating with the response of the patient to therapy. AFP was assessed by standard clinical tests. The DAPI^+^/ICG^+^/GPC3^+^ cell number shown is an average of counts by the standard and imaging flow cytometers for the HB83 sample. The DAPI^+^/ICG^+^ cell number shown was measured by the standard flow cytometer. CT images from the time of TARE and the time of hepatectomy with tumor (red arrows) decrease shown. (B, C) Validation of GPC3^+^ and ICG^+^ primary samples from patients. (B) Histology of primary patient tumor sample showing positivity of sample for GPC3. The scale bar represents 100 μm. (C) Near-infrared imaging of ICG^+^ lung nodule during metastasectomy. (D) Image of ICG^+^/GPC3^+^/DAPI^+^ CTC from Amnis ImageStream instrument. Abbreviations: AFP, alpha-fetoprotein; bf, brightfield; CTC, circulating tumor cell; GPC3, Glypican-3; ICG, indocyanine green; TARE, transarterial radioembolization.

For 4 patients, we had data from at least 2 clinical encounters, and analyses of these samples showed that CTC burden correlated with overall patient response to therapy. This is shown for 1 very-low-risk patient in Figure 4, 2 high-risk patients in Figure 5, and 1 patient with HCC in Figure 6.

We analyzed 2 samples from the HB70/HB110 patient, 1 from the time of hepatectomy when the patient had not yet received chemotherapy and 1 from a follow-up clinical appointment (17.5 months after hepatectomy) when the patient was deemed free of disease (Figure 4). This patient was diagnosed with a very-low-risk PRETEXT I lesion and was treated with upfront resection followed by 2 cycles of C5V (cisplatin, 5-fluorouracil, and vincristine) adjuvant chemotherapy. Currently, this patient is in remission. This patient was strongly positive for both GPC3 (Table 2 and Figure 4B) and ICG (Figure 4C). During that time, CTC burden dropped from 31.8 cells/mL to 11 cells/mL and AFP levels dropped from 978 to 3.5, corresponding to the response of the tumor cells to therapy (Figure 4A). Shown in Figure 4D is an image of a DAPI^+^/ICG^+^/GPC3^+^ CTC analyzed by the Amnis imaging flow cytometer from the sample from the follow-up clinical encounter.

We analyzed 2 samples from the HB102/HB106 high-risk patient, who was diagnosed with a nonmetastatic PRETEXT III HB tumor with HCC features and was treated with 3 rounds of chemotherapy, followed by transarterial radioembolization (TARE) and then resection. After surgery, the patient received adjuvant pembrolizumab but then died due to disease. We analyzed 1 sample from when the patient received TARE and 1 from the time of hepatectomy surgery (Figures 5A–D). This patient showed low, heterogeneous GPC3 expression (Table 2 and Figure 5B) and a strongly positive ICG signal (Figure 5C). During this time, although the patient was receiving chemotherapy, the tumor cells did not respond and the disease burden was increasing. This was assessed based on standard clinical readouts for disease response measured during that time. For example, there was no change in the AFP level (Figure 5A). In addition, the tumor removed with resection was 30% viable. We also measured an increase in CTC burden from 7.03 cells/mL at the time of TARE to 49 cells/mL at the time of hepatectomy (Figure 5A). Furthermore, when we reanalyzed our flow data for only DAPI^+^/ICG^+^ cells, we observed even more CTCs at both time points (Figure 5A). Shown in Figure 5D is an image of 2 DAPI^+^/ICG^+^/GPC3^+^ CTCs analyzed by the Amnis imaging flow cytometer from the sample at the time of hepatectomy.

We also analyzed 2 samples from the HB103/HB113 high-risk patient who was initially diagnosed with a high-risk PRETEXT IV HB tumor and treated with 3 cycles of cisplatin/doxorubicin chemotherapy followed by transplantation and then completion of carboplatin/doxorubicin chemotherapy. The tumor removed during surgery was >99% viable. The patient was then deemed to be disease-free based on no disease shown by imaging and normal AFP levels. After 8 weeks of routine follow-up, the patient was found to have a rising AFP and relapsed metastatic disease by surveillance imaging. They then underwent 6 cycles of vincristine/irinotecan and staged resection of lung tumors. During the first metastasectomy surgery, 1 nodule was identified by CT before surgery (Figure 5E, left CT images), and 2 more were found by ICG during surgery; all 3 were removed and were positive for the disease. During the third metastasectomy surgery, 3 nodules had been identified by CT (Figure 5E, right CT images), and 8 more were identified during surgery by ICG; all 11 nodules were removed, and 3 were positive for tumor. Despite receiving all of this therapy, the patient passed away. We analyzed 2 blood samples from this patient, both from metastasectomy surgeries after relapse. The patient was strongly positive for both GPC3 (Table 2 and Figure 5F) and ICG (Figure 5G). At the first surgery, the patient only had detectable tumor cells in the left lung (Figure 5E, left CT images) and a low AFP of 52.6; at this time point, we did not detect CTCs. From the second sample, we detected 19.5 cells/mL, which corresponds to the rise in AFP to 601. We also analyzed the samples for DAPI+/ICG+/GPC3^-^ cells and found that most ICG^+^ cells were GPC3^+^, consistent with the immunohistochemistry results for GPC3 (Figure 5F). Shown in Figure 5H is an image of a DAPI^+^/ICG^+^/GPC3^+^ CTC analyzed by the Amnis imaging flow cytometer from the sample from the second metastasectomy.

We analyzed 3 samples from the HB83/HB114/HB116 patient with HCC who was initially diagnosed with a PRETEXT IV metastatic HB. They received 2 cycles of cisplatin/5-fluorouracil/vincristine induction chemotherapy and moderately responded with AFP decrease and shrinkage of masses by imaging, including resolution of metastatic lesions in the right lung. They then progressed and underwent a right metastasectomy in an effort to qualify for liver transplantation. The metastatic lesion was determined to be HCC, so they were transitioned to HCC-specific therapy and were treated with (1) GEMOX (gemcitabine hydrochloride/oxaliplatin)/sorafenib, (2) TARE, (3) atezolizumab/bevacizumab, (4) IL15-GPC3 chimeric antigen receptor T cells, (5) vincristine/irinotecan, (6) lenvatinib, and (7) pembrolizumab. They responded to pembrolizumab, which resulted in a significant AFP decrease and reduction of the primary lesion to allow liver tumor and lung metastasis surgeries. In the metastasectomy, they had a total of 15 lesions removed and only 1 was still viable for disease. This patient is still alive and receiving treatment. We obtained 3 samples from this patient, the first from the time of TARE after multiple rounds of chemotherapy when there was a high disease burden, the second from hepatectomy after the patient responded to phase 1 experimental therapy, and the third at the time of metastasectomy (Figure 6A). This patient was strongly positive for both GPC3 (Table 2 and Figure 6B) and ICG (Figure 6C). The first sample showed a higher CTC burden of 8.75 cells/mL, corresponding to a very high AFP of >207,000 (Figure 6A). The tumor responded to therapy as indicated by standard clinical measures, and the CTC burden dropped to 0.2 cells/mL and AFP to 10.5 (Figure 6A). At the third time point, the CTC count increased to 8 cells/mL while the AFP continued to drop to 2.1 (Figure 6A). We also analyzed the samples for DAPI+/ICG+/GPC3^-^ cells and found that there were many ICG^+^ cells that were GPC3^−^ in the first sample, which was not consistent with the GPC3 immunohistochemistry (Figure 6A). Shown in Figure 6D is an image of a DAPI^+^/ICG^+^/GPC3^+^ CTC analyzed by the Amnis imaging flow cytometer from the first sample during TARE.

## DISCUSSION

The major challenge in the field of liquid biopsy for CTCs has been how to unambiguously identify these rare cells for research and clinical purposes. Initial work used epithelial markers to distinguish CTCs that arose from carcinomas. In fact, the only FDA-approved method for CTC enumeration for clinical care is the CELLSEARCH platform, which relies on such tumor markers to identify CTCs.^21^ However, it has become clear more recently that CTCs from carcinomas may undergo an epithelial mesenchymal transition as they intravasate into vessels, thus losing their epithelial markers.^41,42^ Thus, other unbiased methods have emerged, including the isolation of CTCs based on physical properties such as size, charge, density, and elasticity.^42^

For liver cancer, particularly HCC, previous work has investigated the use of a number of technologies for CTC quantification, including CELLSEARCH, CanPatrol, CTC-Chip, ISET, and flow cytometry.^43^ CELLSEARCH, CTC-Chip, and flow cytometry methods rely on marker expression of cells, while ISET technology depends exclusively on cell size and CanPatrol combines size and marker-based techniques. Our work using a fluorescent dye that specifically accumulates in liver tumor cells is similar to other marker-based methods without relying on the expression of a specific gene or protein. Instead, we employed the use of a dye that stains all tumor cells independent of gene or protein expression, which is particularly important for pediatric liver cancer that presents as a very heterogeneous tumor with few markers common to all tumors. We chose to also use GPC3 in our panel to identify tumor cells because it is established to be one of the only proteins elevated in both HB^35^ and HCC.^36^ Finding a common marker is particularly challenging because these tumors are composed of cells of different lineages; HB is an embryonal tumor with both epithelial and mesenchymal areas^1^ while HCC is composed only of cells with an epithelial origin.^44^ Thus, other, more well-established cancer markers are not specific to both of these tumors.

Importantly, analyses of ICG and GPC3 positivity from intraoperative imaging and histology, respectively, validated and informed our use of ICG and GPC3 to identify CTCs. All patients tested were strongly positive for ICG, confirming our use of ICG as the main marker for identifying liver tumor cells. By and large, patients who showed low or heterogenous GPC3 expression also showed the presence of CTCs that were DAPI^+^/ICG^+^ but GPC3^−^. These data show that crosstalk between surgeons and pathologists is key to determining how our CTC panel is analyzed. Future studies should focus on substituting additional markers for GPC3 for GPC3^−^ tumors, particularly markers that may be useful for monitoring FLC. This study also shows that our test is highly sensitive and can be consistently used, even in the absence of GPC3 expression. In our initial experiments to examine ICG accumulation in liver tumor cells, we also observed residual ICG signal in the lung cancer cell line A549 by flow cytometry, which supports previous reports that lung cancers accumulate ICG.^31,32^ This discrepancy in ICG accumulation of A549 cells between the microscopy and flow cytometry experiments is due to different sensitivities of the 2 methods. Our test using ICG to identify CTCs may be more widely applicable to other tumors that accumulate ICG, and our panel could be easily adapted to these tumors by substituting GPC3 with alternative tumor-specific markers.

The second overarching problem to address is how to integrate CTC levels into clinical care. Most studies evaluating outcomes associated with CTC levels for disease monitoring have been focused on common adult cancers including colorectal, breast, and genitourinary tumors.^18,45–47^ A key clinical trial with 778 patients with hormone receptor-positive, ERBB2-negative metastatic breast cancer (STIC CTC METABREAST trial) showed that separating patients into chemotherapy or endocrine therapy based on CTC count instead of physician’s choice improved median progression-free survival by 5.6 months.^48^ By and large, these studies have established that the presence of CTCs is associated with a worse prognosis in adult patients and that patients with elevated CTC count are likely to benefit from more aggressive therapies. With these common adult solid tumors, large numbers of patients can be quickly evaluated to draw clear conclusions regarding how numbers of CTCs can predict therapeutic responses and outcomes. There is not yet sufficient data available for rare tumors, such as pediatric solid tumors, to inform the potential clinical use of CTC levels in these patients. In the area of pediatric solid tumors, most liquid biopsy work has focused on neuroblastoma and sarcomas.^49^ While the presence of CTCs has been established to be associated with worse outcomes for both cancers, most studies have instead focused on outcomes associated with circulating tumor DNA.^49^ Given that the presence of CTCs has been well established to be associated with poor prognosis in many other solid tumor types, our liquid biopsy protocol may provide this bridge to clinical translation for pediatric liver tumors. In our small study, we were not able to correlate CTC numbers with any patient characteristics or outcomes, likely because our patients represented a range of diagnoses, stages and risk groups, and other disease attributes. Expansion of this study with additional patients will allow us to examine CTC counts in a wider range of patients, for example, at diagnosis or relapse, and determine if levels may be useful to predict outcomes for pediatric patients with liver cancer.

Our data most convincingly show that changes in CTC burden over time while a patient is receiving therapy correlate with the patient’s response to therapy and may even contribute additional information that is not provided by other clinical assays. Notably, CTC burden seems to be a better readout of response for the patient with HCC (HB83/114/116, Figure 6), who showed an increase in CTCs between hepatectomy and metastasectomy procedures when it was clear that they still had disease present by imaging; during the same timeframe, AFP levels for this patient dropped from 10.5 to 2.1. For patient HB102/106, we observed a rising CTC burden between TARE and hepatectomy procedures when tumor burden was also rising; during this same timeframe, AFP levels for this patient had already reached the maximum measurable level and, therefore, provided no further information about the response. Taken together, these data show that our test may be most useful for comparing CTC burdens in sequential blood draws from the same patient during therapy to determine whether tumors are responding. This conclusion is limited by the fact that this study only included 4 patients with serial blood samples. Future work will focus on collecting and analyzing diagnostic blood samples for baseline patient information that we can use to compare further counts from specimens collected during the course of therapy.

In our validation of our panel with noncancer control samples, as well as 1 blood sample from a nonmalignant mesenchymal hamartoma tumor, we showed that this assay detects no more than 1.2 DAPI^+^/ICG^+^/GPC3^+^ cells per mL of blood, proving the specificity and sensitivity of the test. This presence or absence of CTCs is invaluable information that can indicate whether a patient is at risk for metastasis. However, this is complicated by the established fact that CTCs have a short half-life^50^ and not all CTCs are capable of giving rise to detectable metastatic disease.^42^ Future work in our lab and others is focused on identifying and targeting these “bad” CTCs that are capable of colonizing organs outside the organ of origin, and this information will also inform markers for more specific CTC tests to identify the most aggressive CTCs.

Taken together, this work shows a novel way to identify CTCs with the far-red fluorescent dye ICG, which is already used clinically and is a compelling example of bedside-to-bench scientific research. Notably, our test can be used for the 2 most common malignant pediatric primary tumors of the liver, HB and HCC, because both tumor cell types accumulate ICG. This work will form a foundation for future studies to establish how CTC numbers can be used in the routine clinical care of these patients.

## Supplementary Material

**Figure:**
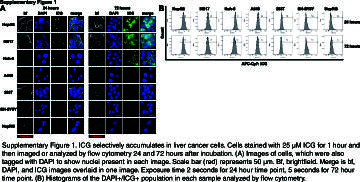


**Figure:**
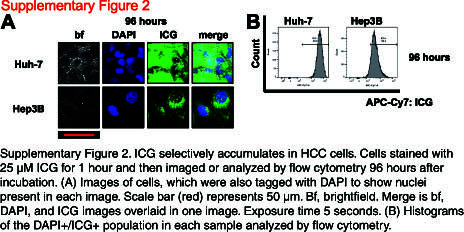


**Figure:**
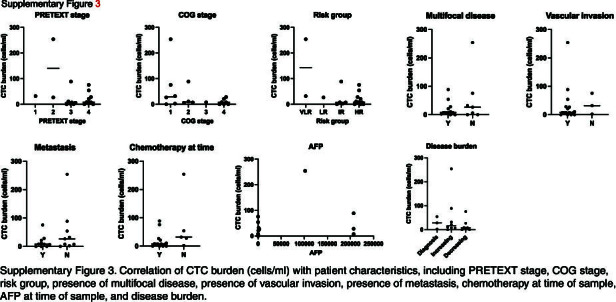


## Data Availability

The data generated in this study are available upon request from the corresponding author.

## STATEMENT OF TRANSLATIONAL RELEVANCE

This manuscript represents the first report of circulating tumor cells in the blood of pediatric patients with liver cancer. The novel and innovative assay for CTCs shown in this paper will facilitate future work examining the relationship between CTC numbers and patient outcomes and serve as the foundation to incorporate liquid biopsy into routine clinical care for these patients.
